# Challenges in accessing sexual and reproductive health services by people with physical disabilities in Kampala, Uganda

**DOI:** 10.1186/1742-4755-11-59

**Published:** 2014-08-02

**Authors:** Sharon Eva Ahumuza, Joseph KB Matovu, John Bosco Ddamulira, Florence Kyoheirwe Muhanguzi

**Affiliations:** 1 MakSPH-CDC Fellowship Program, Makerere University College of Health Sciences, School of Public Health, P.O. Box 7072, Kampala, Uganda; 2 Department of Disease Control & Environmental Health, Makerere University College of Health Sciences School of Public Health, Kampala, Uganda; 3 Makerere University College of Humanities and Social Sciences, School of Women and Gender Studies, Kampala, Uganda

**Keywords:** Persons with physical disability, Sexual and reproductive health services, Challenges in accessing sexual and reproductive health services

## Abstract

**Introduction:**

Despite the universal right to access the same range, quality and standard of free or affordable health care and programs as provided to other persons, people with physical disabilities (PWPDs) continue to experience challenges in accessing these services. This article presents the challenges faced by PWPDs in accessing sexual and reproductive health (SRH) services in Kampala, Uganda.

**Methods:**

This was a qualitative study that was conducted with male and female PWPDs in Kampala in 2007. Data on the challenges experienced by PWPDs in accessing SRH services were collected using in-depth interviews with 40 PWPDs and key informant interviews with 10 PWPDs’ representatives, staff of agencies supporting PWPDs and health workers. All data were captured verbatim using an audio-tape recorder, entered into a Microsoft Word computer program and analyzed manually following a content thematic approach.

**Results:**

The study findings show that PWPDs face a multitude of challenges in accessing SRH services including negative attitudes of service providers, long queues at health facilities, distant health facilities, high costs of services involved, unfriendly physical structures and the perception from able-bodied people that PWPDs should be asexual.

**Conclusion:**

People with physical disabilities (PWPDs) face health facility-related (service provider and facility-related challenges), economic and societal challenges in accessing SRH services. These findings call for a need to sensitize service providers on SRH needs of PWPDs for better support and for the government to enforce the provision of PWPD-friendly services in all health facilities.

## Background

The United Nations Convention on the Rights of Persons with Disabilities [1] and other international human rights conventions [2] guarantee the fundamental human rights to physical, social, and psychological health. Specifically, the UN Convention on the Rights of Persons with Disabilities guarantees persons with disabilities the right to access “the same range, quality and standard of free or affordable health care and programs as provided to other persons, including those in the area of sexual and reproductive health and population-based public health programs” [1]. However, available evidence suggests that persons with disabilities (PWDs) still face numerous challenges in accessing and utilizing essential health services [3] and this affects their quality of life [3,4]. Impediments to receiving the required health services include attitudinal biases of health and social service providers, physical barriers in clinical settings, and poor dissemination of information [5,6]. Persons with disabilities also experience lack of privacy and respect by health interventions in addition to various aspects of their care needs not being acknowledged [5,7,8].

In a study on the health care access and support for disabled women in Canada, Gibson and Mykitiuk [9] found that health system policies and practices reflect erroneous assumptions about what disabled people can or should do and that disabled women were often discouraged from having children either because of doubts regarding their capacities to provide care or because of concerns regarding the risks of the child inheriting a hereditary condition. These findings suggest that despite the call for universal access to reproductive health services at the 4^th^ International Conference on Population and Development in Cairo in 1994 [10] and the right to access “the same range, quality and standard of free or affordable health care and programs as provided to other persons” [1], access to sexual and reproductive health (SRH) services by PWDs remains a critical challenge. Albert and Hurst [11] attribute people with disabilities’ inability to access health services to a complex web of discrimination made up of negative social attitudes and cultural assumptions as well as environmental barriers including policies, laws, structures and services which result in marginalization and social exclusion. Access to SRH services by PWDs is also hampered by inaccessible health facilities, insensitivity of health care providers, limited knowledge by health care providers about disability, and limited information tailored to their health needs [5,11]. In general, the reduced status of PWDs and the equation of sexuality to being normal and not disabled [1] add up to an orientation towards the denial of SRH services to PWDs [12].

However, while previous studies have explored the factors affecting access to and utilization of health services by PWDs in general [7,13] or specific population groups such as women with disabilities [9], few studies have explored the challenges faced by persons with physical disabilities (PWPDs) in accessing SRH services. This presents a missed opportunity for understanding the challenges that these people go through in accessing SRH services, and impedes our ability to assist them to enjoy the same health services that able-bodied persons enjoy. In this study we bridged this gap by exploring the challenges faced by male and female persons with physical disabilities in accessing SRH services in Kampala, Uganda.

## Methods

### Study design

This was a qualitative study that explored the challenges that PWPDs face in accessing SRH services in Kampala, Uganda. Data collection took place in 2007. The choice of the qualitative approach was based on the fact that this approach provides the opportunity [14] to document lived experiences of PWPDs in accessing SRH services through more in-depth and detailed narratives. The qualitative approach made it possible to understand the social processes associated with accessing SRH services and the challenges encountered in accessing these services from the point of view of PWPDs themselves [14,15]. SRH services were defined as services related to antenatal care, child-birth services, and access to sexual and reproductive health information.

### Study setting

The study was conducted in Kampala, Uganda’s Capital City. The selection of Kampala as the study site was based on the fact that many of the agencies that advocate for PWDs’ rights have head offices here (see names of these agencies below), in addition to the fact that it would be cost-effective to contact PWDs who come to the City to seek opportunities for self-employment or being employed by the above-mentioned agencies, and invite them for participating in this study.

### Participants’ recruitment and data collection

We approached potential participants with the help of two leading agencies supporting PWPDs in Uganda; National Union of Disabled Persons in Uganda (NUDIPU) and Uganda National Action of Physical Disability (UNAPD). These two agencies linked us to one of the associations of PWPDs (Kampala Business Association for Disabled People) from where the first participant was contacted and invited to participate in the study. After interviewing this participant, he led the team to another participant who was also interviewed. These two participants then acted as “seeds” that led us to their counterparts in a process of snowball sampling. Those identified were approached for participation in the study and the trend continued until the required number for participants was interviewed. A total of 40 PWPDs (20 female and 20 male) aged 18 years and above were interviewed, including the two “seeds” contacted initially. The in-depth interviews with PWPDs captured information relating to the socio-demographic and economic characteristics (age, sex, religion, marital status, age at marriage, education level, type of employment, main source of income, means of transport and number of children), membership to associations and sources of information on SRH, and challenges faced by PWPDs in relation to accessing to SRH services.

In addition to in-depth interviews, one key informant (usually the officer responsible for SRH services) was selected purposively from each of the organizations which advocate for PWPDs’ rights in Uganda (all these organizations have offices in Kampala) including NUDIPU, Action for Disability, Ministry of Gender Labor and Social Development (MoGLSD); Ministry of Health (MoH), Federation of Uganda Women Lawyers (FIDA), and the Uganda Police Force forming a total of six key informants. An additional four participants were drawn from Mulago National Referral Hospital (1); Kampala Business Association for Disabled People (2) and Parliament of Uganda (1) (Member of Parliament representing people with disabilities). While the intention was to interview an equal number of male and female key informants (5 male and 5 female), this was not possible because most of the staff responsible for SRH and PWPDs were male. Consequently, 8 male and 2 female key informants were interviewed. Key informant interviews focused on the perceived challenges faced by PWPDs in accessing SRH services as well as suggestions for addressing them. Interviews were conducted in English and *Luganda*, the local language, for those who did not understand English. Qualitative data were captured verbatim using an audio-tape recorder and entered into a Microsoft Word computer program prior to analysis.

### Data analysis

To facilitate comparative analysis of challenges encountered by male and female PWPDs, responses to structured questions on background characteristics of respondents and challenges encountered were coded and entered into EpiData and analyzed using SPSS version 17 to generate frequency tables. Printed transcripts of qualitative data were analyzed manually using the content thematic approach, which was guided by the Graneheim and Lundman frame work [16]. At the time of analysis, SEA and FKM independently read through the interview scripts and coded the text following pre-determined themes and sub-themes, and met to compare notes. Intra-coder differences were noted and resolved through checking the main transcripts while emerging themes were discussed and either adopted or rejected. The major theme was challenges encountered by PWPDs in relation to SRH services. This theme was used to code data from interview scripts. Sub-group analysis was conducted, which involved examining the theme in relation to male and female PWPDs as well as service providers. This was intended to identify the unique and cross-cutting challenges encountered by PWPDs in their endeavor to access SRH services. Direct quotations were identified and used in presentation of study findings.

### Ethical consideration

Approval for the study was granted by the Department of Women and Gender Studies, the Faculty of Social Sciences and School of Graduate Studies Makerere University. Permission to carry out the study was obtained from NUDIPU, UNAPD and other organizations involved in the care and advocacy for PWPDs. Given that issues of disability and SRH are very sensitive, the researcher always introduced the purpose of the study to participants. Participants who agreed to participate in the study signed consent form or put a thumbprint for those who were illiterate. Participation in the study was voluntary and participants were assured that anonymity would be observed at all times. Confidentiality of participants was maintained by using numbers on the transcripts. Of all the PWPDs approached for the interview, only one declined to be interviewed because of unfulfilled promises by past researchers who promised assistance that was never realized.

## Results

### Socio-demographic characteristics of PWPDs

Table 1 shows the characteristics of the 40 PWPDs who were interviewed for this study stratified by gender. Compared to females, a higher proportion of male PWPDs were older (e.g. 60% of males were aged 36 years and above as opposed to 10% of females), currently married (male: 65%; female: 35%) and formally employed (male: 95%; female: 65%). On the other hand, a higher proportion of females were better educated (75% of female PWPDs had secondary or higher levels of education compared to 65% among male PWPDs) and engaged in small businesses than their male counterparts (female: 95%; male: 70%). Similarly, a higher proportion of female PWPDs (65%) reported ‘small businesses’ as their main source of income compared to 55% among male PWPDs. An equal proportion of both males and females (i.e. 70%) reported that they were members of associations of disabled persons in Kampala.

**Table 1:** Socio-demographic characteristics of PWPDs in Kampala, Uganda

**Characteristic**	**Male**		**Female**	
	**n = 20**	**%**	**n = 20**	**%**
Age				
18-24	1	5	5	25
25-30	6	30	8	40
31-35	1	5	5	25
36+	12	60	2	10
**Marital status**				
Single	5	25	11	55
Married	13	65	7	35
Just living together	1	5	2	10
**Level of education**				
No education	1	5	0	0
Primary	6	30	5	25
Secondary	6	30	7	35
Tertiary	7	35	8	40
**Currently Employed**				
Yes	19	95	13	65
No	1	5	6	30
**Type of employment**				
Informal employment (Small business)	14	70	19	95
Formal employment	6	30	1	5
**Main source of income**				
Formal employment	6	30	1	5
Small Business	11	55	13	65
Relatives	0	0	6	30
Other	3	15	0	0
**Membership to association**				
Yes	14	70	14	70
No	6	30	6	30

### Challenges encountered by PWPDs in accessing SRH services

Table 2 shows the type of challenges experienced by PWPDs in accessing SRH services stratified by gender. Poor physical accessibility, negative attitudes of health workers and long queues at the health facilities were singled out as the main challenges that PWPDs face while trying to access SRH services in Kampala. Other challenges included long distances to the health facilities, high costs of services, and the fact that health workers are not experienced to handle PWPDs or even fear them. Important to note is the fact that female PWPDs experienced these challenges at a higher level compared to their male counterparts (and this is true across all challenges). For instance, while only 55% of males reported poor physical accessibility as a challenge to accessing SRH services, the proportion of females who reported this challenge was 80%. Similarly, while only 50% of male PWPDs reported negative attitudes of health workers as a challenge to accessing SRH services, the proportion of females who experienced this challenge was 75%. The following sub-sections present detailed narrations of the experiences and challenges that PWPDs experience as they try to access SRH services. These challenges have been grouped into five main challenges: poor physical inaccessibility, negative health workers’ attitudes; long queues at the health facilities, economic challenges (i.e. high cost of services) and marginalization/social discrimination.

**Table 2:** Challenges encountered by PWPDs in accessing SRH services in Kampala, Uganda

**Challenges encountered in accessing SRH services by PWPDs (proportion reporting ‘Yes’)**	**Male**		**Female**	
	**n = 20**	**%**	**n = 20**	**%**
Poor physical accessibility	11	55	16	80
Negative attitude of health workers	10	50	15	75
Long queues	07	35	09	45
Long distance to health facility	06	30	08	40
High costs of services	05	25	07	35
Health workers not experienced to handle/fear PWPDs	02	10	06	30

Multiple responses were allowed.

### Physical inaccessibility

As shown in Table 2, physical inaccessibility was the major challenge experienced by both male and female PWPDs in trying to access SRH services because of the unfriendly physical facilities. As indicated in the quotations below, most PWPDs pointed out that most health care facilities lack ramps; personnel to assist PWPDs such as helping them climb stairs; wheel chairs and disability friendly beds in case of delivery or admission:

“Almost all the health facilities in our midst have steps and therefore moving upwards to other levels is very hard for us. For example, much as Mulago is the national referral hospital and with qualified medical staff, it is not easy for us to access…” (Female PWPD)

*“Even if you have money, if you are an expecting woman who has physical disabilities particularly us with hip joints or round legs, you may never give birth from the private wing on 6*^*th*^*floor which is the safest level to deliver from in Mulago hospital. Climbing there is not easy” (Female PWPD)*

“I would have liked to accompany my wife during the ANC visits, but it is just too hard for me to climb stairs. Even when health workers are to assist, they usually ignore us (men). May be we are not expected to go there” (Male PWPD)

Consequently pregnant women seeking antenatal and child birth care services have difficulties in accessing these services. The limited access to health facilities characterized by lack of ramps coupled with unfriendly facilities such as labor beds and separate toilets for PWPDs in health units was confirmed through key informant interviews with technical staff at health facilities and Ministry of Health officials as illustrated in the following voice.

“The PWPDs are not well catered for in our hospitals. Labor wards lack special facilities for PWPDs such as adjustable delivery beds and health centers are not easily accessed due to many steps and lack of ramps. Although suggestions have been made on the need to make health care facilities disability friendly, the Ministry of Health has not yet put [this] into consideration” (Key informant)

The lack of appropriate and friendly facilities for PWPDs is a clear manifestation of the marginalization of PWPDS’ SRH health needs. Furthermore, marginalization and vulnerability of PWPDs is illustrated in the reported attitudes of health workers, which were noted to be negative characterized by abusive language used when attending to mothers who come for antenatal care (ANC) and delivery at the health facilities. One of the female respondents noted;

“For me when I went for ANC when I was pregnant health workers said, “even you in your status you sleep with men and more so you accept to conceive? Men do not forgive; imagine they also sleep with this disabled woman”” (Female PWPD)

#### Negative attitudes of health care providers

As shown in Table 2, health care providers’ negative attitude is the second most important challenge that PWPDs face while accessing SRH services. This is usually compounded by societal beliefs and expectation that PWPDs should not conceive at all:

“You know it is like women with physical disabilities should not conceive at all. When I go for pregnancy checkup, the way midwives look at me, is like I have done something wrong! At times they are too rude to me but I have learnt to ignore them and just aim at getting someone to check the condition of my baby. We do not like the way society and health care providers treat us” (Female PWPD)

“One time I tried to seek for SRH information but the nurse told me that it was not useful for me since in my condition [as a man with physical disabilities], chances of even impregnating a woman seemed limited according to her thinking” (Male PWPD)

Key informant interviews with health care providers yielded the same sentiments, suggesting that negative health workers’ attitudes is a major barrier to PWPDs’ access to SRH services:

“I know when PWPDs get pregnant, they are despised, not expected to conceive due to the assumption that they already have enough problems to deal with. This happens mostly with health care providers” (Key informant)

These findings indicate that PWPDs particularly women suffer from societal stigmatization and blankness under the pretext that they should not become pregnant and give birth owing to their disability. The female PWPDs were concerned about constant reminders on how they should be asexual and abuses related to their appearance which they noted to cause stigma and de-motivation from using health facilities. The above voices illustrate that PWPDs particularly females are expected by society to be concerned more about their disability rather than SRH matters.

Both PWPDs and key informants indicated that health facilities are ill-prepared to address the SRH needs of PWPDs. Most respondents mentioned that health care providers were not trained to handle PWPDs, and that some health care providers subject females with physical disabilities to deliver by cesarean section, thereby minimizing their ability to deliver normally. This is particularly due to lack of skills to handle pregnant females with disabilities, as the following quotations illustrate:

“Service providers are not trained in special skills to handle PWPDs. Health care providers get shocked when they receive pregnant PWPDs at health facilities. This should not be the case…” (Key informant)

“We need to appreciate that delivering PWPDs requires particular skills and surely we do not have them at the moment…..” (Key informant)

#### Long queues at health facilities

Long queues in the health facilities pose particular vulnerability to PWPDs, whose condition as opposed to the able-bodied clients may not stand the waiting time as these female respondents noted;

“If it was not lining up in health centers, I believe more women with physical disabilities would be going there for services. At times when I would go for ANC services, I would line up for a long time and sometimes I would get so tired and give up. Our hip bones are not strong enough to stand for a long time and when we are pregnant we tend to feel weak and tired most of the time” (Female PWPD)

“What puts me off is that when I go for antenatal care and I am told to wait until they call my number. I do not have a wheel chair and I cannot sit on the bench. I have to sit on the floor” (Female PWPD)

The long queues lead to loss of time and getting tired which is also exacerbated by the physical limitations associated with physical disability and lack of positive discrimination by the health workers where PWPDs are left to line-up with those who do not have disabilities.

#### Economic challenges/high cost of services

Inaccessibility to SRH services was also associated with the high cost of services. Most respondents noted that the services were costly and that many PWPDs could not afford them given the meager incomes that they earn:

“It is not enough to know that you have a right to utilize health care during delivery yet you cannot afford it when you need it. For sure most of the PWPDs have little income and cannot afford health care; their health rights remain on paper” (Key informant)

“In the better hospitals, health workers cannot attend to us unless if we pay yet charges are so high. Most of us PWPDs resort to giving birth from small health facilities which are not expensive. Even when you do not have all the required money, health workers there are patient with us” (Female PWPDs)

The practice of paying for health services even at public health facilities where such services are expected to be free was a common concern in the narratives of PWPDs. Some PWPDs who could afford to pay, mentioned that they had relatively easier access to health care than those who could not afford.

#### Marginalization/social discrimination of PWPDs

Marginalization in the provision of SRH services was mentioned as one of the challenges that afflict both men and women with physical disabilities as they try to access services. One of the male respondents lamented about the treatment he received at the health facility as he escorted his spouse for ANC and delivery services.

“I went to hospital with my wife for pregnancy checkup and because we all have physical disabilities, only my wife was helped to climb steps. I was told they did not need me and so whatever was done on my wife, I was not informed. I could not even get someone to address my concerns as a husband expecting a baby” (Male PWPD)

Narratives from male and female PWPDs revealed that it was common for males to be told to leave or to wait from outside while their wives are being attended to. This left males with unanswered questions and concerns regarding to the care their wives and babies would need. Societal marginalization of PWPDs is also reflected in the way they are treated as they travel to seek care. Given their low economic status, most PWPDs in this study used public transport – i.e. taxis – with few having their own private cars. Among the respondents only 4 (10%) out of the 40 participants used their private cars to travel to seek care. The encounter with public transport was described as a nightmare for PWPDs characterized by marginalization by both taxi operators and fellow passengers as some respondents explained;

“…Even you in your condition with a pregnancy, what are you going to do in town?” (Female PWPD)

“As a woman was boarding a taxi that was heading to the ANC center, she heard other women complaining loudly ‘…Bakateyamba bajja kutulwisa’ literally meaning persons who cannot help themselves will delay us” (Female PWPD)

Some community members also assume that PWPDs do not have sexual interests. Self-pity and the desire to conform to societal expectation among PWPDs further constrain access to SRH services.

“Most PWPDs know where to find these services but at times they fear to be seen seeking them because the rest of the society thinks we do not need these services. For example people do not expect a PWPD to contract HIV because they assume we are not sexually active. Society forgets that we are normal human beings with feelings as well” (Key informant)

Both male and female respondents noted inherent societal expectations and misperception that PWPDs do not need SRH services including SRH information. Consequently, there is overprotection of children with physical disabilities especially girls through denial of information as the following quotation illustrates:

“Girls with physical disabilities are so much protected by their care takers who assume that they will not engage in sex. They are usually kept away from discussions about sex and reproduction at home and in communities. Such PWPDs usually get this information when it is too late usually at health facilities” (Key informant)

In such situations, PWPDs miss out on opportunities to get health information that could help them to make informed decisions. Such denial of information increases PWPDs vulnerability to SRH problems.

## Discussion

Our study of the challenges faced by PWPDs in accessing SRH services in Kampala, Uganda showed that PWPDs encountered health facility-related, economic, and societal challenges. Physical inaccessibility, negative attitude of health care providers and long queues formed one main cluster of challenges while cost of services and marginalization coupled with negative societal expectations formed another cluster of challenges. Majority of the PWPDs were engaged in small businesses, suggesting that they earned low incomes which are not enough to support their livelihoods as well as pay for the cost of health services. This was particularly true among female PWPDs who experience majority of the challenges, partly because of their role as mothers and partly because a high proportion of them (compared to males) were engaged in small businesses. These findings suggest a need to address health facility-related challenges as well as provide PWPDs – and particularly female PWPDs – with viable income generating activities that could boost their earnings. The findings also suggest a need to sensitize the general public about SRH needs and rights of PWPDs including educating the general public that save for their physical disability, PWPDs are as normal as other able-bodied people and should therefore have equal access to the same services enjoyed by able-bodied persons.

With regard to physical inaccessibility, study findings revealed that PWPDs’ access and utilization of SRH services in Kampala was constrained by lack of appropriate physical facilities such as ramps, adjustable beds especially in labor wards, wheel chairs and disability-friendly sanitation facilities in hospitals. These constraints have also been highlighted by WHO and UNFPA as key health facility barriers to PWPDs’ access to SRH services [17]. These findings concur with those of Gipson [18] who argues that PWPDs’ ability to gain entry to health services is influenced by architectural and transportation considerations among others. Nteere [19] in a study on SRH rights in Kenya noted that PWPDs are never included in platform of advocacy for SRH services and government plans.

The physical inaccessibility dimensions revealed in our study are in part, a reflection of inherent shortcomings and marginalization of PWPDs within the planning and design of health facilities in Uganda. Our findings contrast SRH rights that are already recognized in international human rights and other consensus documents [1,20]. At national level, our findings on physical inaccessibility contrast the commitment by the government through the Uganda constitution to guarantee universal access to health as a human right [21]. In addition, inaccessibility of health facilities emerged as a challenge despite the availability of the Persons with Disability (PWD) Act of Uganda that advocates for access to buildings by PWDs including provision of ramps, safe and accessible sanitation facilities and adequate railing around hazardous areas [22]. The implication here is that whereas the Government of Uganda strives to increase access to health facilities by reducing physical distance, our findings indicate that reducing physical distances to the health facilities is not enough to guarantee accessibility to services by PWPDs. It is thus imperative that physical disability-friendly facilities such as ramps, adjustable beds, wheel chairs and sanitation are provided in all existing and future health facilities. The continued physical barriers to PWDs access to health services, indeed constitutes social exclusion that requires more efforts to ensure that policies formulated are implemented to deliver real benefits to PWPDs.

Negative attitude of health care providers emerged a key challenge to PWPDs’ access to SRH services in Kampala. This was reflected in the way PWPDs were treated at the health facilities including use of abusive language by health care providers. The negative attitude of health care providers was linked to the assumption that PWPDs should be asexual, especially women who were not expected to become pregnant. Our finding on attitudinal biases of health care providers as a challenge to PWPDs’ access to health services concur with what has been documented in other settings [5,6]. In Uganda, negative attitude of health care providers towards PWPDs remains a reality despite the constitutional and PWD Act provisions that seek to ensure that persons with disabilities enjoy the same rights with other members of the public in all health institutions, and that health professionals provide care of the same quality to persons with disabilities as to others [21,22].These findings affirm that realization of SRH as a human right by PWPDs requires fostering positive attitude among health care providers to appreciate the needs of PWDs particularly those with physical disabilities. For this to become a reality, training of health care providers is essential. In this regard, care for PWPDs integrated in pre-service as well as in-service training of health workers should be strengthened.

In addition, PWPDs waited in long queues at health facilities in their struggle to access SRH services. This is partly attributed to few health workers. For instance, the 2011 Uganda Ministry of Health Sector Performance Report indicated that only 56% of positions of health workers were filled [23]. Whereas long queues is a common occurrence especially at public health facilities in Uganda [24], lack of consideration for persons with physical disability was a hindrance to access of SRH services by PWPDs. These findings in part reflect inadequate attention to fostering affirmative action in favor of PWDs as provided for by the PWD Act [22].

Our findings further reveal that societal perceptions that PWPDs should not be sexually active influence the way PWPDs are treated in the community on issues of SRH. The social perceptions contribute to the marginalization of PWPDs as society sees no need of such services for them; they are often abused and insulted publicly in taxis and other public spaces. Such marginalization restricts their movement and hence limits PWPDs’ access to SRH services. Restrictions on SRH information for PWPDs with the belief that they should not be sexually active constrained PWPDs’ access to SRH services. This has also contributed to withholding of sex education on the assumption that PWPDs ‘won’t need it’. Such withholding of sexuality information limits PWPDs’ opportunities for learning about SRH. Our findings concur with those of Cole [25] who noted that parents of disabled girls are often overprotective and withhold information about SRH. Consequently, PWPDs miss out on opportunities to learn about SRH as they are perceived to have enough challenges to deal with rather than SRH issues [26]. The discriminatory practices illustrate the ways in which the sexuality of PWPDs and their dignity is demeaned and dishonored by the negative attitudes and lack of attention by different actors in society. The negative societal attitude was compounded by the desire by some PWPDs to conform to societal expectation that they are asexual. Thus, efforts geared at improving PWPDs’ access to SRH services should include activities aiming at changing the negative societal attitude towards PWPDs’ sexuality issues, but also building self-esteem among PWPDs to demand and use SRH services.

Study finding show that male PWPDs experienced discrimination and marginalization in relation to SRH from service providers and the public. Findings further demonstrate that SRH has been perceived as a female affair and health workers were not trained to handle the needs of PWPDs more so male PWPDs. The common practice of male discrimination reported in this study, contradicts the ongoing efforts and interest to promote male involvement in maternal and child health [27,28]. For instance the programme for prevention of mother-to-child transmission of HIV in Uganda seeks to increase male partners counseled and tested for better results [29].Whereas some men with physical disabilities made an effort to overcome the multiple barriers that they encounter and accompanied their sexual partners to health facilities, such men were in general not attended to by health care providers. On the contrary, these men were instead discriminated against based on their gender and physical disability. Our findings challenge reproductive health actors and advocates to address the unique male related vulnerabilities among PWPDs in the broader efforts to realize male involvement in child and maternal health in Uganda.

The findings of this study should be interpreted in light of a number of limitations. We enrolled a limited number of PWPDs in Kampala, thus these may not be representative of all persons with disabilities in Uganda. It is likely that the challenges faced by urban-based PWPDs might be different from those experienced by PWPDs living in rural areas, an area that warrants further study. In addition, given the differences in accessibility to health facilities between urban and rural areas, inaccessibility of health facilities could be more pronounced for PWPDs in rural areas. The qualitative nature of the study also limits the generalization of findings beyond the study setting. However, the exploratory nature of the study facilitated an in-depth understanding of PWPD’s lived experiences, largely consistent with what has been documented elsewhere. Thus, study findings may have wider applicability beyond the study area.

Nevertheless, our study present important findings that highlighted the challenges that PWPDs face in accessing SRH services in Kampala, Uganda. While these findings may not be representative of the challenges faced by all PWPDs, they create a window of opportunity for provision of SRH services to these largely marginalized populations in an urban setting. The findings help to highlight the need for improving access to SRH for PWPDs as well as changing health workers’ attitudes towards them. This is in line with the international statutes such as; the UN Convention on the Rights of Persons with Disabilities, the Universal Declaration of Human Rights; the Convention on elimination of all forms of discrimination against women that guarantee universal access to SRH services [20,30-32].

## Conclusion

In conclusion, access to sexual and reproductive health services by PWPDs in Kampala was limited by a multitude of challenges ranging from health facility-related, economic and societal challenges. These findings suggest a need for government and other stakeholders to ensure that policy provisions that improve access to SRH services by PWPDs are actualized. The findings reveal the need to equip health workers with knowledge and skills to enable them adequately address the needs of PWPDs at health care centers, and to devise alternative interventions to address the plight of males with physical disabilities.

## Abbreviations

ANC: Antenatal care; HIV: Human immunodeficiency syndrome; MoGLSD: Ministry of Gender, Labor and Social Development; MOH: Ministry of Health; WHO: World Health Organization; NUDIPU: National Union of Disabled Persons in Uganda; UNAPID: Uganda National Action of Physical Disability; PWD: Persons with Disability; PWPD: Persons with physical disabilities; SRH: Sexual and reproductive health; RH: Reproductive health; UN: United Nations; UNFPA: United Nations Fund for Population Activities; FIDA: Federation of Women Lawyers.

## Competing interests

The authors declare that they have no competing interests.

## Authors’ contributions

SEA: Made substantial contributions to conception, design, data collection, analysis and interpretation of data, wrote the first draft of the paper and was responsible for the final submission of the paper. JKBM: made substantial contributions to conception, design and interpretation of data, contributed to drafting of the paper, revised the drafted paper for important intellectual content and approved the version to be published. JBD: Revised the draft paper for important intellectual content and approved the version to be published. FKM: Made substantial contributions to conception and design, acquisition of data, analysis and interpretation of data, contributed to the drafting of the paper, revised the draft paper for important intellectual content and approved the version to be published. All authors reviewed and approved the final manuscript for submission.

## Authors’ informations

SEA is a Social Scientist with a Master’s degree in Women and Gender Studies and is a MakSPH-CDC Fellow attached to AIDS Information Center; JKBM is a Behavioral Scientist with a Master of Health Science degree in International Health. He is the Training Manager for the MakSPH-CDC Fellowship Program at Makerere University College of Health Sciences, School of Public Health, Kampala, Uganda; JBD is a Medical Doctor, with a Master’s degree in Public Health and is a Lecturer at the Department of Disease Control & Environmental Health Sciences, Makerere University College of Health Sciences, School of Public Health, Kampala, Uganda; FKM is a Social Scientist with a Master’s in Demography, a Doctor of Philosophy degree in Gender Studies and is a Senior Lecturer at the School of Women and Gender Studies, Makerere University College of Humanities and Social Sciences, Kampala, Uganda.
